# Adult-Specific Systemic Over-Expression Reveals Novel *In Vivo* Effects of the Soluble Forms of ActRIIA, ActRIIB and BMPRII

**DOI:** 10.1371/journal.pone.0078076

**Published:** 2013-10-21

**Authors:** Kengo Yamawaki, Shinobu Ueda, Tsutomu Okada, Takeshi Oshima, Makoto Kakitani, Takashi Kato, Kazuma Tomizuka

**Affiliations:** 1 Biologics Research Laboratories, Kyowa Hakko Kirin Co., Ltd, Machida-shi, Tokyo, Japan; 2 Comprehensive Research Organization, Institute for Innovation Design, Waseda University, Shinjuku-ku, Tokyo, Japan; 3 Department of Biology, School of Education, Waseda University, Shinjuku-ku, Tokyo, Japan; 4 Kyowa Hakko Kirin California, Inc., La Jolla, San Diego, California, United States of America; Children's Hospital Los Angeles, United States of America

## Abstract

Bone morphogenetic proteins (BMPs)/growth differentiation factors (GDFs), which belong to the TGF-beta superfamily, are pleiotropic factors that play a role in regulating the embryonic development and postnatal homeostasis of various organs and tissues by controlling cellular differentiation, proliferation and apoptosis. Conventional transgenic and knockout (KO) mouse approaches have provided only limited information regarding the *in vivo* functions of BMP signaling in adult animals due to the effects on prenatal development and the difficulty in manipulating multiligand signals simultaneously. We recently produced transgenic chimeric mice(Tg chimeras) in which the soluble IgG1-Fc fusion protein of three BMP type II receptors (ActRIIA, ActRIIB, BMPRII) was highly circulated (281-709 μg/ml), specifically in adult mouse blood. Since each BMP receptor can bind to multiple BMP ligands, these Tg chimeras should be useful to investigate the effects of trapping multiple BMP ligands. Remarkably, some phenotypes were unexpected based on previous studies, such as KO mouse analyses, presumably representing the effects of the multiple ligand trapping. These phenotypes included increased red blood cells (RBCs) and decreased viability in adults. In a further study, we focused on the phenotype of increased RBCs and found that extramedullary hematopoiesis in the spleen, not in the bone marrow, was increased using histological and flow cytometric analyses. Although it remains to be elucidated whether the transgene products affect the tissues directly or indirectly, our data provide novel and important insight into the biological functions of the soluble IgG1-Fc fusion protein of three BMP type II receptors in adults, and our approach should have broad applications to research on other ligand receptor families and studies involving mouse models.

## Introduction

Bone morphogenetic proteins (BMPs)/growth differentiation factors (GDFs), secreted proteins that belong to the TGF-beta superfamily, are pleiotropic factors that play a variety of roles in the regulation of embryonic development and postnatal homeostasis of various organs and tissues by controlling cellular differentiation, proliferation and apoptosis [1,2]. In mammals, there are more than 20 BMP/GDF ligands, seven type I receptors (Activin receptor-like kinase (ALK) 1-7) and five type II receptors (ActRIIA, ActRIIB, BMPRII, TGFβRII, MISIIR). The BMP/GDF ligands transduce signals through a complex of type I and II serine/threonine kinase receptors via Smad-dependent and -independent cascades that eventually evoke the transcriptional activation of downstream target genes. To elucidate the physiological functions of BMP/GDF ligands and their receptors, phenotypic analyses of knockout (KO) mice have been widely employed [3-7]. For example, muscle hypertrophy in GDF8 (myostatin) KO mice [8], the abnormal formation of the heart in BMP2,4 and 10 KO mice [9-11], atrophy of the testes in BMP4, 7 and 8a KO mice [12,13], increased serum levels of iron in BMP6 KO mice [14], increased levels of bone and hematopoietic stem cells in BMPRIA conditional KO mice [15] and increased pulmonary artery vascular permeability in BMPRII conditional KO mice [16] have been reported as observed phenotypes. Although these studies have provided valuable information regarding the non-redundant functions of each BMP/GDF ligand and their receptor, there is still only limited knowledge regarding how each BMP signaling pathway works independently or synergistically to regulate a variety of biological processes in the living body, in which many ligands and receptors coexist. In such a context, recently published studies generating and analyzing compound KO mice [17-19] represent a significant advancement toward understanding the physiological role of this complex ligand/receptor family. 

Ligand trapping with soluble receptors is considered to be an alternative “loss of function” approach to studying the effects of the blockade of signaling mediated by ligand/receptor interactions. Furthermore, etanercept, a soluble form of the tumor necrosis factor receptor, is one example of the therapeutic application of this strategy. Many type I and type II receptors can bind to BMP ligands by themselves [20-23]. For example, the known ligands for three type II receptors (ActRIIA/B and BMPRII) are BMPs (BMP2,3,4,5,6,7,8,9,10,15), GDFs (GDF1,3,5,6,7,8,9,10,11), Activin-A/B, Inhibin-A/C/E and Nodal. Therefore, the extracellular domains of BMP receptors fused with the immunoglobulin Fc region have often been used to investigate the effects of blocking BMP signaling *in vitro*. Recent studies have also described the effects of the administration of ActRIIA-Fc and ActRIIB-Fc in mice, including increased bone volume and muscle hypertrophy [24-26]. Since *in vitro* studies have indicated that BMP receptors can bind to multiple ligands with very diverse affinity [20-22], the phenotype observed in mice administered ActRIIA-Fc and ActRIIB-Fc likely represents the effects of blocking a limited number of relatively high-affinity ligands. Although the serum concentrations of the soluble receptors administered in these studies have not been reported, we hypothesize that maintaining a very high level of circulating soluble BMP type II receptors in mice could allow for the simultaneous trapping of more ligands, including relatively low affinity ligands, thereby providing new information regarding the biological functions of BMP signaling.

We previously reported a novel transgenic chimeric mouse system that can be used to rapidly evaluate the functions of genes encoding secreted proteins. Briefly, we used the immunoglobulin kappa (Igk) promoter and inserted the transgene units into the genomic site adjacent to the endogenous Igk locus of the murine embryonic stem (ES) cell using homologous recombination. The resultant ES clones were injected into embryos derived from a B-cell-deficient host strain, thus producing a chimerism-independent, B-cell-specific transgene expression [27]. In the transgenic chimeric mice (Tg chimeras) produced using this system, the target secreted proteins are expressed specifically in adults and highly circulated in the blood. Using this system, we are the first group to successfully identify the potent and specific proliferative effects of R-spondin1 on intestinal crypt cells [28]. 

In the present study, we made great improvements to the system and produced three kinds of Tg chimeras in which soluble forms of three BMP type II receptors (ActRIIA, ActRIIB, BMPRII) are highly circulated as Fc fusion proteins in the blood and conducted a comparative analysis of the resulting phenotypes. We identified some unexpected phenotypes based on previous studies, such as KO mouse and soluble receptor administered mouse analyses, that are common to all three Tg chimeras, including increased red blood cells (RBCs), extramedullary hematopoiesis in the spleen and decreased viability in adults. These phenotypes presumably represent the effects of simultaneous multiple ligand trapping. Therefore, our data should provide novel and important insight into the biological functions of BMP signaling in adults, and our approach should have broad applications to research on other ligand receptor families and studies involving mouse models.

## Results and Discussion

### Generation of Tg chimeras highly expressing transgenes

**Figure 1:**
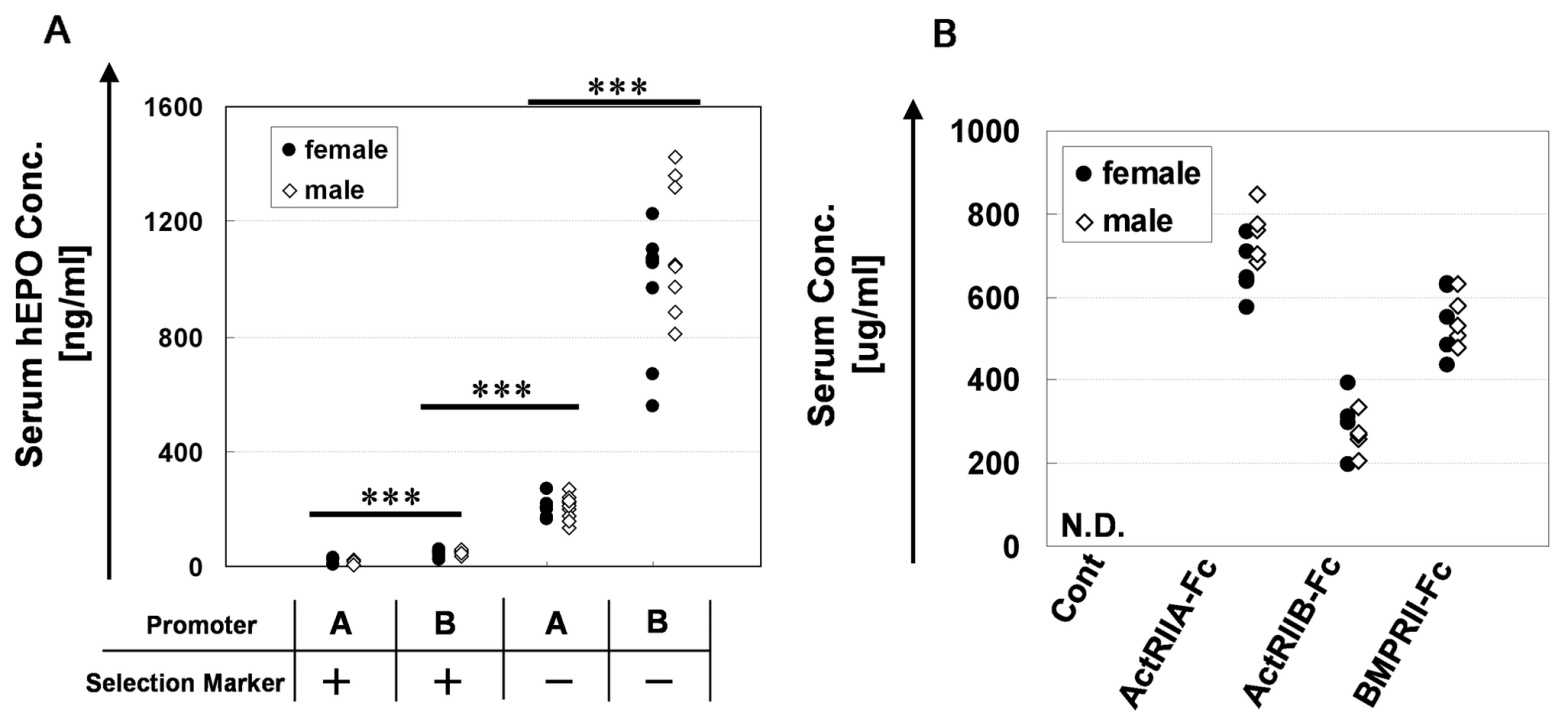
Serum protein levels produced from the transgenes in each Tg chimeras. A. Serum hEPO concentrations of the four types of eight-week-old Tg chimeras identified using a combination of two different promoters (A: used in our previous report, B: newly developed for this study) and the existence of puro and neo selection markers. ***, P<0.001 (Student’s *t*-test). Significant differences were observed in both females and males. B. The serum concentration of each soluble protein (ActRIIA-Fc, ActRIIB-Fc and BMPRII-Fc) in 11- to 16-week-old Tg chimeras was measured using ELISA (see the Materials and Methods). In the control chimeras, the Fc protein was not detectable. As described in Materials and Methods, the “control” chimeras are produced using ES cells in which the expression unit is not introduced.

To achieve a much higher transgene expression in chimeric mice, regarding the promoter and leader sequence, the modifications described below, which differ from the method described in our previous report, were applied (1). Using a promoter derived from a different Igk variable region (Igkv3-12, 0.5kb) from that used in a previous report (Igkv4-54, 0.21kb) (2), a leader sequence located downstream of the described promoter (1) was used (3). Although the native leader sequence of the transfer gene was used in our previous report, we artificially joined the leader sequence of (2) to the transfer gene from which the native leader sequence coding region had been removed in this study. In addition to the above change, the puromycin-resistant gene cassette located downstream of the transfer gene and the neomycin-resistant gene cassette located in the RS region were removed using the Cre/loxP system [27] (Figure S1). Figure 1A shows the serum human erythropoietin (hEPO) concentration in eight-week-old hEPO-introduced Tg chimeras that were generated using these modifications (change in promoter and leader sequences and excision of drug resistant markers). Each modification showed an apparent effect that increased the hEPO expression level, and notably, the combination of both improvements resulted in a ~200-fold increase in the hEPO expression level. Although further studies are required to clarify the molecular mechanisms underlying this effect, it is noteworthy that the presence of selection markers upstream of the Igk 3’ enhancer may suppress the expression of the immunoglobulin kappa gene [29]. By applying two types of modifications on the original method, we were able to generate a series of Tg chimeras which have different levels of transgene expression. This will be a great advantage for the in vivo analyses of gene functions because it will help to avoid the lethality caused by inappropriate expression and will facilitate evaluations of the relationship between the expression level of the target gene and the resulting phenotype [30].. 

To investigate whether the Tg chimeras produced according to our method could be used to analyze the ligand-trap effect of Fc-fusion proteins *in vivo*, we generated Tg chimeras expressing the EPO receptor (EPOR)-Fc and first analyzed the hematological parameters of these mice. At 8 weeks of age, the average serum concentration of EPOR-Fc was 24.7 μg/ml, and the mice exhibited severe anemia (Table S1). Therefore, these results suggested that, at least from the age of 8 weeks, the ligand-trap effect was achieved in the Tg chimeras produced using our approach.

We generated three kinds of Tg chimeras expressing ActRIIA-Fc, ActRIIB-Fc and BMPRII-Fc using the highest expression type generated with the improved transgenic system. The average serum concentration of each of the soluble proteins in 11- to 16-week-old Tg chimeras measured using ELISA was 709, 281 and 548 μg/ml, respectively (Figure 1B). As mentioned in our previous report, the expression level of the transgene was uniform in each mouse, independent of the coat color chimerism (range: 10%-100%).

### Phenotypic analysis of the Tg chimeras

**Figure 2:**
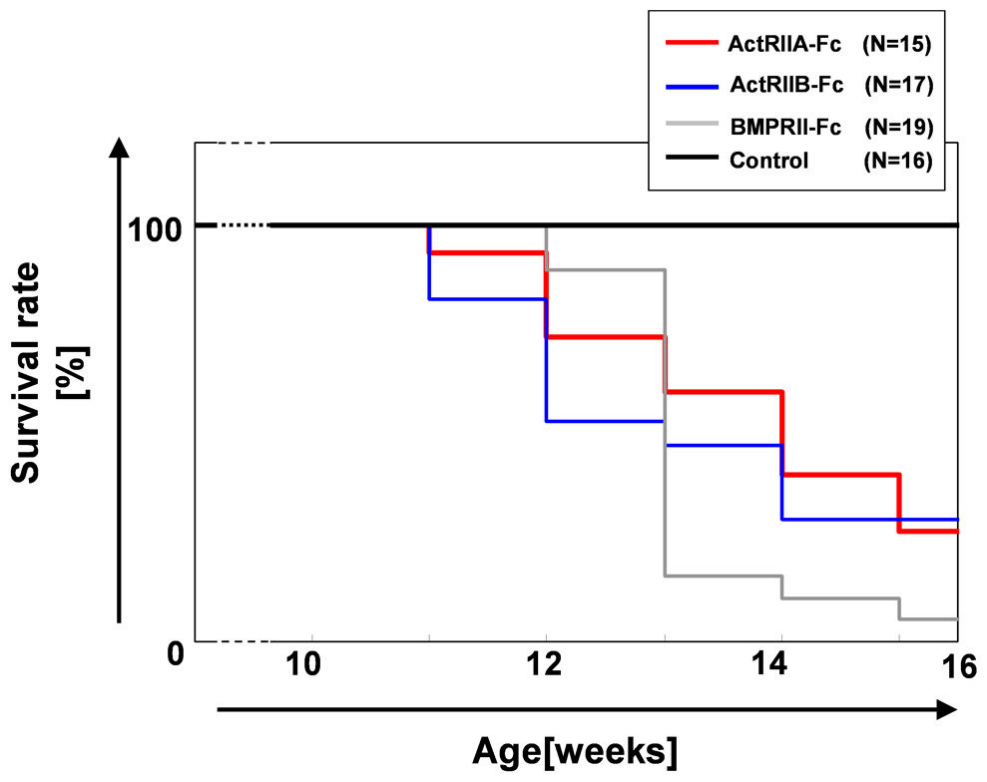
Survival rate of each Tg chimeras. The term before 10 weeks was abbreviated because all chimeric mice were still alive.

**Figure 3:**
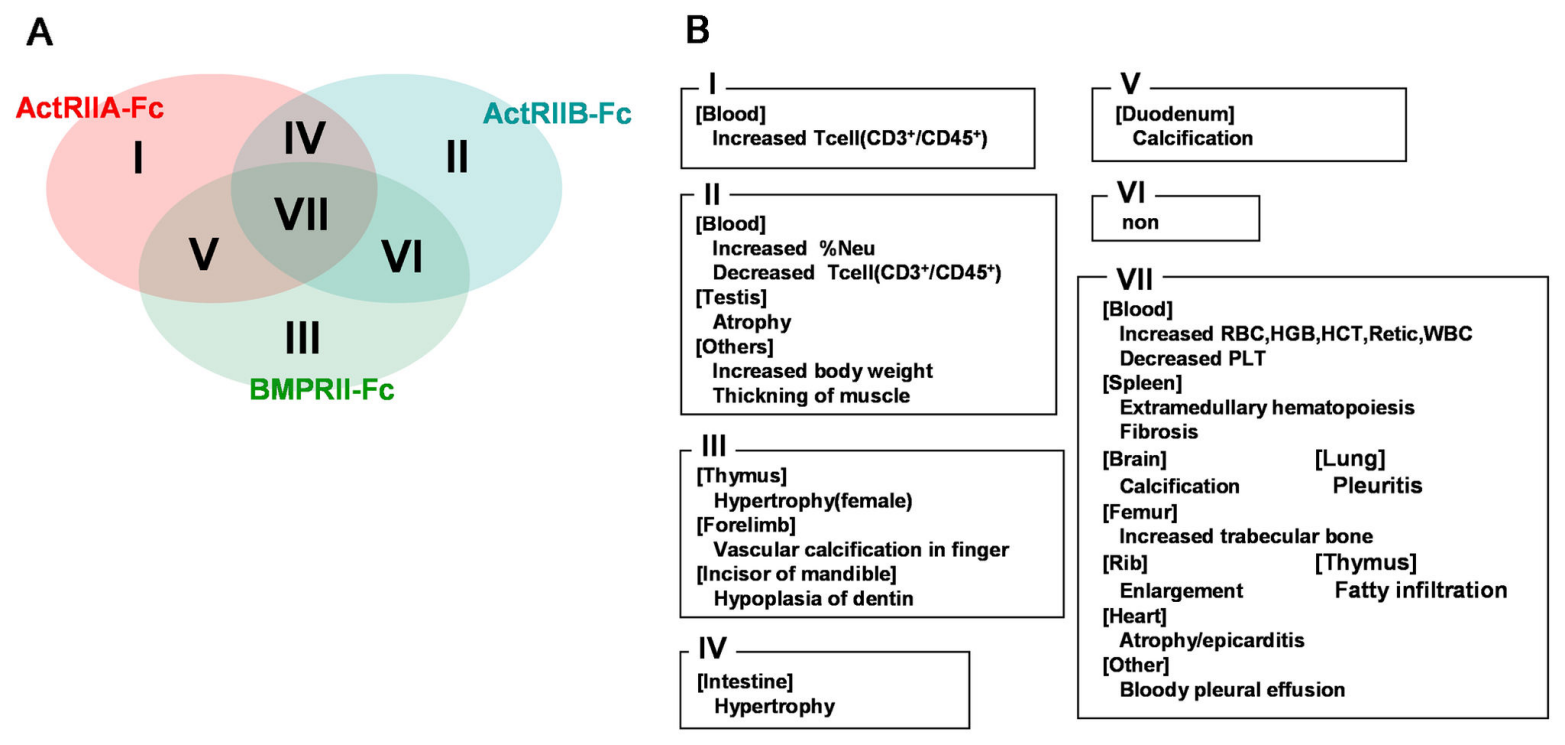
Summary and grouping of the phenotypes observed in each Tg chimeras (11- to 16 weeks old). A. Venn diagram representing the common phenotypes of the three Tg chimeras. The ellipses represent the phenotypes obtained from each Tg chimeras. The common phenotypes are shown in the overlapping sections. B. Summary of the phenotypes. The notes correspond to the data shown in Figure 3A. For example, VII means that the features were common to all three (ActRIIA-Fc, ActRIIB-Fc and BMPRII-Fc) Tg chimeras.

A summary of the phenotypes of the three kinds of chimeric mice is shown in Figures 2 and 3. The Tg chimeras exhibited poorer general health than the control chimeras for each of the transgenes (ActRIIA-Fc/ActRIIB-Fc and BMPRII-Fc) from 11 weeks, and the survival rates at 16 weeks were 27%, 29% and 0% (including the immediately necropsied mice), respectively (Figure 2). Comparing the phenotypes of the three kinds of chimeric mice (11-16 weeks old), we found groups of phenotypes that were common among the three Tg chimeras (increased RBCs, atrophy of the heart, calcification of the brain, increased trabecular bone, bloody pleural effusion, etc.), common among two Tg chimeras (hypertrophy of the intestines, calcification of the duodenum, etc.) and particular to different Tg chimeras (increased T-cells, muscle hypertrophy, hypoplasia of dentin in the incisors of the mandible, etc.)(Figures 3A,B). To determine whether those phenotypes are due to the effect of hFc or not, we also generated the Tg chimeras that expressed only hFc (serum concentration was 218 μg/ml) and performed the phenotype analysis as described in “Phenotype analysis of Tg chimeras” in materials and methods. There was no obvious phenotype in those Tg chimeras (data not shown). 

We cannot exclude the possibility that some of observed phenotypes are indirect effect resulted from the BMP-ligand trapping. However, brain calcification, increased trabecular bone, extramedullary hematopoiesis in spleen and increased RBCs were also clearly observed at 8weeks-age (other tissues were not analyzed), and these phenotypes observed in younger mice could represent direct effects of transgene products. Nevertheless, time course of phenotypic analysis and evaluation of the change in BMP signaling in affected tissues should be necessary to determine whether those phenotypes are direct effect of the BMP-ligand trapping.

On the other hand, our approach that enables systemic and long-term exposure of proteins to mice would be useful in terms of toxicological assessment of recombinant protein therapeutics. Early death was not reported in a previous study in which ActRIIA-Fc and ActRIIB-Fc were administered in mice [24-26,31]. Although the serum concentrations in the mice were not reported, it is likely that a longer term and higher levels of circulation of the soluble forms of the proteins were achieved in the chimeric mice in our study. That is, we believe that the blockade of multiple BMP ligands and their signals by the high levels of the soluble forms of the proteins in circulation may have caused the death in adult. ActRIIA-Fc is now undergoing clinical trials for the treatment of anemia. Our results suggest that ActRIIA-Fc possibly induces adverse effects when it is circulated at a high concentration over the long term. Therefore, maintaining optimum control of the serum concentration is very important in order to ensure the safety of ActRIIA-Fc. In addition, these findings are considered to be a good example showing that the multi-trap strategy can lead to the occurrence of novel *in vivo* effects. In other words, our system can be used to screen drug candidates whose effects are due to multiligand trapping.

Although the direct cause of death remains unclear, it is worth noting that “bloody pleural effusion” was observed in all three kinds of chimeric mice. It has previously been reported that BMP signaling is related to vascular homeostasis. For example, haploinsufficiency of BMPRII and ALK1 underlies the pathogenesis of pulmonary arterial hypertension (PAH) and hereditary hemorrhagic telangiectasia (HHT), respectively [32-34], and in BMPRII KO and knockdown (KD) mice, pulmonary vascular leakage and bleeding, respectively, are observed[16,35]. In each of these reports, it was mentioned that endothelial dysfunction due to the disturbance of BMP signaling may be the cause of these phenotypes. At least two BMP family ligands, BMP9 and BMP10, are capable of signaling through a receptor complex formed by ALK1 and BMPRII [36-38]. These two ligands may also have an affinity for ActRIIA, ActRIIB and BMPRII [20,39]. Although it is difficult to conclude whether the “bloody pleural effusion” was caused by endothelial dysfunction due to the blockade of BMP9 and 10 signaling based only on the phenotype, our findings provide a basis for further studies to clarify the molecular mechanisms underlying this phenomenon.

Previously, *in vivo* phenotypes, such as increased trabecular bone induced by the administration of ActRIIA-Fc [24] as well as muscle hypertrophy and increased trabecular bone induced by the administration of ActRIIB-Fc, have been reported [25,26]. Our Tg chimeras expressing ActRIIA-Fc and ActRIIB-Fc also exhibited the same phenotypes (Table 1, Figures S2 and S3). These results suggest that the functional soluble form was affected in the Tg chimeras, confirming the validity of our system.

**Table 1:** Hematological parameters of each Tg chimera.

		**N**	**age**	**RBC(^10^6^[cells/ul])**	**HGB([g/dL])**	**HCT([%])**	**Retic (^10^9^[cells/L])**
**ActRIIA**	**female**	**6**	**8w**	13.6 ± 0.88^***^	20.2±1.12^***^	64.3±3.52^***^	458.2±76.0^***^
	**male**	**6**	**8w**	13.4 ±1.17^***^	19.9±1.20^***^	64.0±3.65^***^	459.4±37.2^***^
**ActRIIB**	**female**	**6**	**8w**	11.7±0.95^**^	18.4±1.19^**^	58.9±3.82^***^	393.8±115.5^**^
	**male**	**5**	**8w**	11.7±0.42^***^	18.4±0.28^***^	59.4±1.73^***^	456.3±96.5^***^
**BMPRII**	**female**	**6**	**9w**	13.0±0.49^***^	19.6±0.98^***^	61.7±2.37^***^	510.9±70.7^***^
	**male**	**6**	**9w**	13.0±0.49^***^	19.8±0.55^***^	62.0±0.72^***^	448.3±49.1^***^
**Cont**	**female**	**16**	**8-9w**	**9.9±0.41**	**15.3±0.41**	**47.7±1.19**	**284.7±48.9**
	**male**	**16**	**8-9w**	**10.1±0.32**	**15.6±0.52**	**48.9±1.50**	**301.4±48.6**

The values are presented as the mean ±SD. ** P<0.01 vs. Cont ; *** P<0.001 vs. Cont. (Student’s *t*-test)

The mouse phenotypes are grouped in terms of commonality in Figure 3. Some phenotypes were also observed in the BMP ligand KO (including conditional KO) mice. For example, abnormal formation of the heart in BMP2, 4 and 10 KO mice [9-11], hypertrophy of the intestines in BMPRIA KO mice [40,41], atrophy of the testes in BMP4, 7 and 8a KO mice [12,13], thickening of the muscle and increased body weight in GDF8 (myostatin) KO mice [8], increased bone in BMP3 KO mice [42] and hypoplasia of the dentin in BMP4 and 7 KO mice [43,44] have been reported. This suggests that our system can certainly achieve ligand-trapping of several BMPs, thereby creating conditions that are equivalent to those observed in KO mice. The phenotypes common to all three Tg chimeras may possibly be attributed to the blockade of ligands that can interact with all three receptors, while the phenotypes unique to each of the Tg chimeras may be attributed to the blockade of ligands with high affinity for specific receptors. A notable example of this hypothesis is the muscle hypertrophy phenotype observed only in the ActRIIB-Fc Tg chimera, which correlates with the findings of a previous report showing that GDF8 (myostatin) has very high affinity for ActRIIB [45,46].

### Histological and flow cytometric analyses of erythroid cells

**Figure 4:**
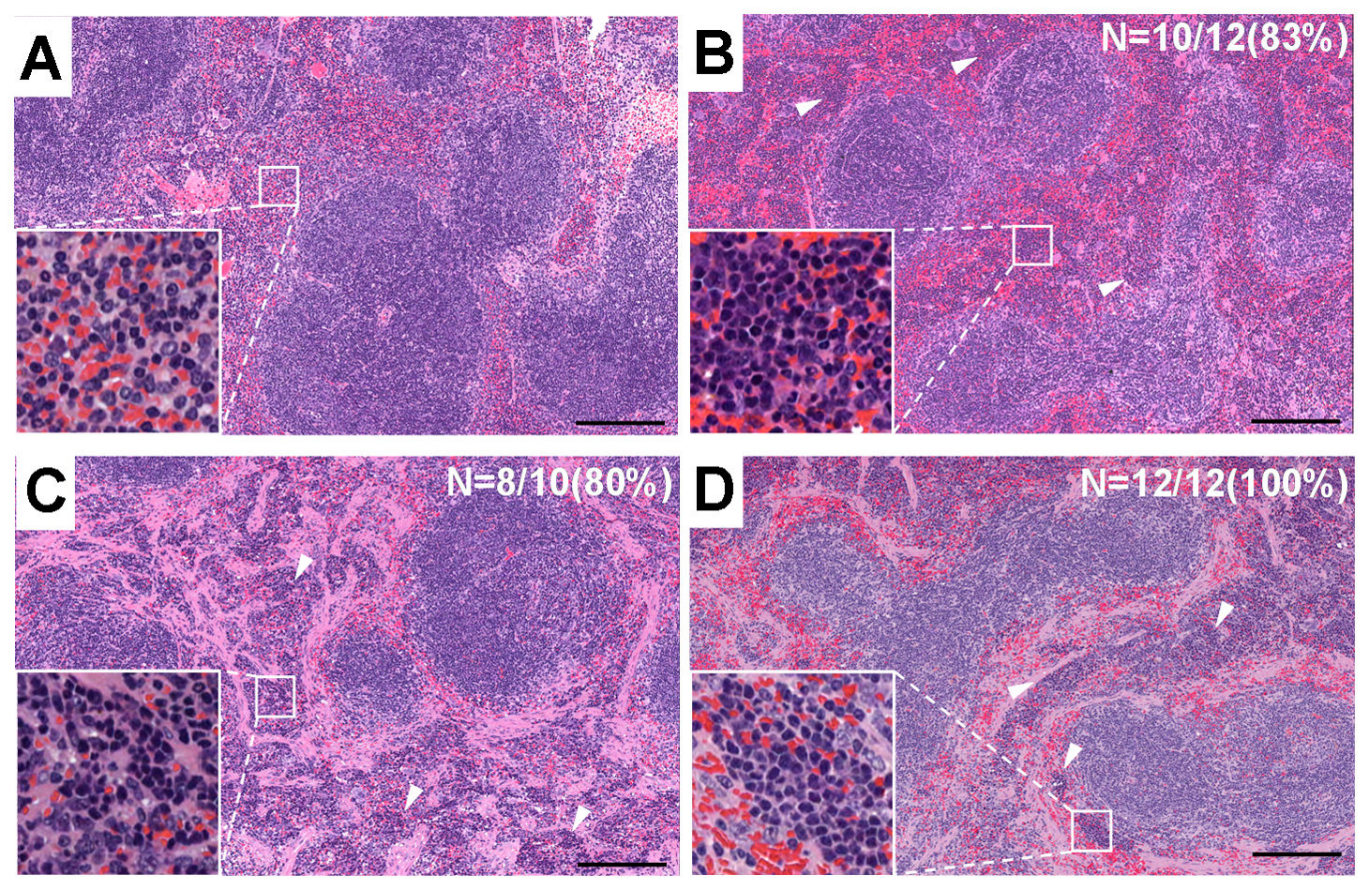
Histological images of the spleen in each group of Tg chimeras (8 weeks old). A. Control, B. ActRIIA-Fc, C. ActRIIB-Fc and D. BMPRII-Fc. As shown in the magnified images, increased extramedullary erythrocytic hematopoiesis was observed. The arrowheads represent foci of erythroblastic islets. Such foci were observed in all three Tg chimeras at a very high frequency (shown as percentages in the figure) but not in the control spleens (only one of the 10 mice exhibited a less severe phenotype). Each scale bar indicates 200 μm.

**Figure 5:**
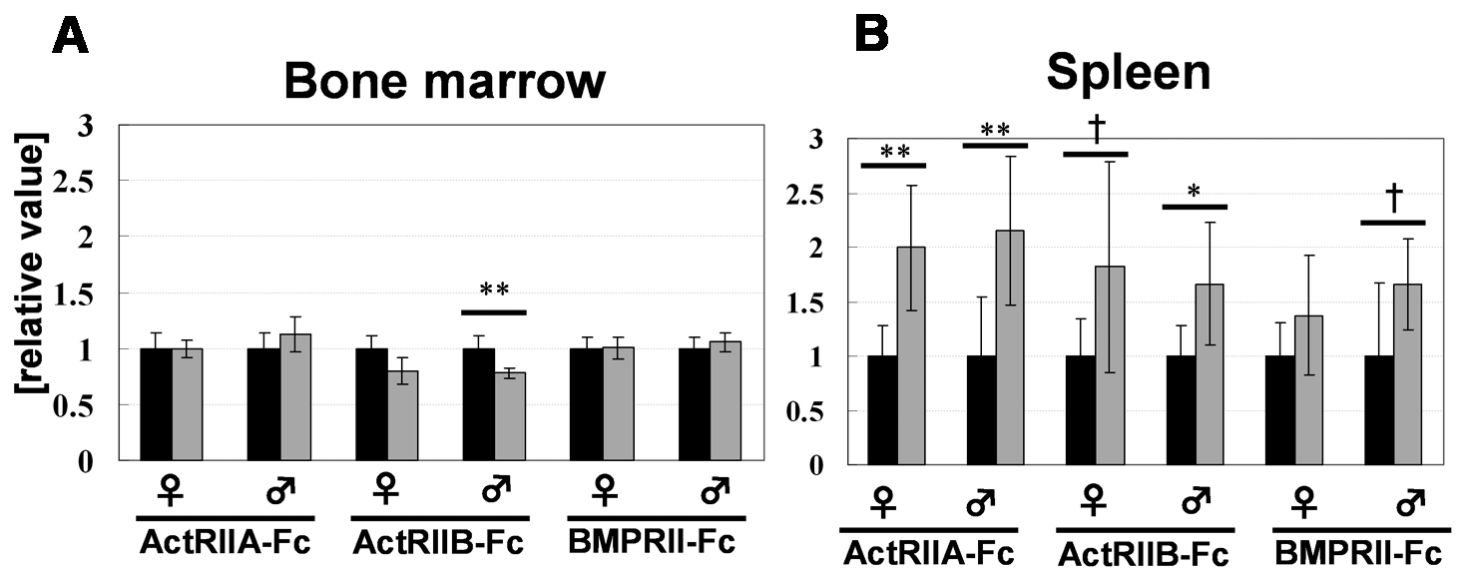
Increased extramedullary hematopoiesis was observed in the chimeric mouse spleens. Results of the flow cytometric analysis of the bone marrow (A) and spleen (B). The black bar indicates the control chimeras and the gray bar indicates the Tg chimeras. Samples were obtained from 8-week-old Tg chimeras (N=6). Each experiment was performed independently, and a control was prepared for each procedure. The ratio of [% of gated cells] in each Tg chimera to that observed in the control chimeras was described as [relative value]. *, P<0.05 ; **, P<0.01; †, tendency (0.05<P<0.10) (Student’s *t*-test).

In this study, we found, for the first time, that ActRIIA-Fc, ActRIIB-Fc and BMPRII-Fc exhibit a biological activity that affects the number of RBCs/Hemoglobin (HGB)/Hematocrit (HCT)/Reticulocyte (Retic) (Table 1). Although it has previously been described that ActRIIA-Fc exerts such effects in a review [31,47], no detailed data to support this claim were shown. Focusing on this phenotype, we performed a histological analysis of the bone marrow and spleens of eight-week-old chimeric mice. Although there were no obvious changes in bone marrow (Figure S4), significant numbers of foci of erythroblastic islets in the spleen were observed in Tg chimeras with a dramatic frequency (Figure 4). Analyzing the CD71+TER119+ erythrocyte lineage distribution of the bone marrow and spleen cells using flow cytometry, an increase in such cells was observed only in the spleen and not in the bone marrow (Figure 5, Figure S5). As mentioned above, this suggests that extramedullary erythrocytic hematopoiesis is increased in all three types of Tg chimeras.

Raje et al. described a mechanism that may be responsible for the increase in RBCs and indicated that blocking activin A may release the inhibition of IL-1b, which subsequently enhances the expression of several erythropoietic growth factors, such as BMP2 and 7 [31]; however, no detailed data were shown. In fact, previous *in vitro* studies suggested that the three type II receptors can bind to activin-A [21,48], and our data indicate that such an effect can also be seen *in vivo*. However, there were no significant changes in the serum IL-1b levels in the Tg chimeras, which suggests that the increased extramedullary hematopoiesis may be related to the upregulation of RBCs. Although a relationship between BMP4 and extramedullary hematopoiesis has been reported [49,50], BMP signaling upregulated extramedullary hematopoiesis in that study, which is in contrast to the results of our study of BMP ligand trapping. This suggests that some changes in body homeostasis are caused by multiligand blocking and that some BMP ligands may negatively regulate extramedullary hematopoiesis. Further studies are thus required to investigate those possibility. 

The BMP/GDF family has diverse and complicated signals and it also plays an important role in development and homeostasis in various animals. Such diversity arises not only from expression patterns, such as timing and tissue specificity, but also from the many interactions between ligands and receptors and the combination of type I-type II receptor complexes [1,51]. Additionally, one receptor can interact with multiple ligands, which is one reason for the complexity [20,52]. Conducting *in vivo* studies is absolutely necessary in order to understand these complicated signaling pathways, because *in vitro* studies do not provide sufficient information.

It is also important to investigate which ligands are trapped and which BMP/GDF-SMAD pathways are indeed regulated in the affected tissues in each mouse. One limitation of the multiple signal interference method is that it is unlikely to contribute deep understanding on the molecular mechanisms of specific BMP ligands. Previous efforts have revealed some ligands that are trapped by ActRIIB-Fc in murine and human sera using proteomic techniques; however, in that paper, the authors also mentioned the difficulty of performing such experiments using the tissue samples [39]. A common approach used to detect the spatial localization of the BMP activity (such as comparison of Smad1/5/8 vs Smad2/3 signaling etc.) is to perform immunostaining of tissues with antibodies. Recently, some groups have pointed out that such an approach can be tedious and time consuming and has the drawback of not sensing the transcriptional response of a cell. These authors reported a transgenic strategy to locally monitor BMP signaling using the BRE-lacZ reporter transgenic strain [53,54]. Such an approach would be very helpful for clarifying the mechanisms underlying the *in vivo* effects obtained in our study. This will be the subject of our future work.

As mentioned above, we analyzed and compared the phenotypes of Tg chimeras that expressed three soluble forms of BMP type II receptors and grouped them according to category, including those possibly due to the blockade of a BMP ligand common to all three receptors, those specific to each receptor, those possibly arising due to multiligand blocking and so on. Additionally, we found, for the first time, that the three soluble forms all led to a significant increase in RBC/HGB/HCT cells and that extramedullary hematopoiesis may be one of the causes of this phenotype. Using our system, the same phenotypic analysis can be performed for all BMP receptors and ligands. Analyses of our results and related previous works would be very helpful for understanding the functions and interactions of BMP ligands and receptors *in vivo*. Furthermore, such studies are not limited to BMP family molecules. The great advantage of this system is that it is a rapid and efficient procedure, while also demonstrating a high-level transgene expression. As a result, it is considered to be a useful tool for elucidating the functions of various secreted proteins, including soluble receptors *in vivo*.

## Materials and Methods

### Generation of Tg chimeras

Chimeric mice expressing hEPO, EPOR-Fc, ActRIIA-Fc, ActRIIB-Fc and BMPRII-Fc fusion proteins were generated as described in ref [27] with certain modifications, i.e., (1) using a promoter derived from a different Igk variable region (Igkv3-12, 0.5kb) from that used in a previous report (Igkv4-54, 0.21kb) (2). A leader sequence located downstream of the described promoter (1) was used (3). Although the native leader sequence of the transfer gene was used in our previous report, we artificially joined the leader sequence of (2) to the transfer gene from which the native leader sequence coding region had been removed in this study. In addition to the above change, the puromycin-resistant gene cassette located downstream of the transfer gene and the neomycin-resistant gene cassette located in the RS region were removed using the Cre/loxP system (see Figure S1 for more information). 

The scheme of chimeric mice production is as follows: The targeted ES cells (C57BL/6 x CBA-F1) were injected into eight-cell to morula stage embryos prepared from the immunoglobulin m heavychain-KO (Dm) homozygous mouse strain (mixed background of C57BL/6 x CBA x MCH(ICR))[27,55]. After development of embryos to blastocysts, approximately 10 injected embryos were transplanted into a pseudopregnant MCH(ICR) mouse. In this study, the “control” chimeras are produced using ES cells in which the expression unit is not introduced. The other scheme is the same as that for the transgene-expressing chimeras. 

The cDNA fragments of the extracellular domains of EPOR (NM_010149.3, 73bp-747bp), ActRIIA (NM_007396.3, 58bp-402bp), ActRIIB (NM_007397.2, 55bp-402bp) and BMPRII (NM_007561.2, 76bp-453bp) were obtained from FANTOM cDNA Clones (RIKEN). 

### Measurement of the serum concentrations of ActRIIA-Fc, ActRIIB-Fc and BMPRII-Fc using ELISA

The serum protein levels produced from the transgenes were measured using ELISA. Serum was obtained from eight-week-old hEPO and EPOR-Fc Tg chimeras and each soluble BMP receptor (ActRIIA-Fc, ActRIIB-Fc and BMPRII-Fc) transgenic chimeric mouse at 11-16 weeks. An R&D Systems Kit was used for hEPO. For each soluble Fc-fusion protein, anti-hIgG (gamma-chain) (SIGMA) was immobilized on the plate and detected with peroxidase conjugated anti-hIgG(Fc)(SIGMA). The hFc(IgG1) was used as a standard. The measured value was recalculated by multiplying the measurement by the ratio of each molecular weight of the soluble form proteins divided by that of the hFc(IgG1). 

### Phenotype analysis of Tg chimeras

All procedures used to measure the items for the phenotypic analysis, such as the hematological parameters, B/T cell population, serum IgG/IgM concentration, blood chemical analysis, organ weight and histopathology (paraffin sections, Hematoxylin and Eosin staining), were performed as described in ref [27]. The mice were sacrificed via exsanguination under ether anesthesia, and all efforts were made to minimize suffering. All procedures were approved by the Institutional Animal Care and Use Committee of Kyowa Hakko Kirin Co. Ltd (Approval Number: A-95). 

### Flow cytometric analysis of erythroid cells

Flow cytometry was performed on a FACS-Aria instrument (Beckton-Dickinson). The isolation and preparation of bone marrow and spleen cells were performed by flushing the femurs and spleens of eight-week-old chimeric mice with PBS. The reagents used in this study were as follows: anti-Ter119-APC (BD), anti-CD71-PE, (BD), Fc block (BD), live or dead (Invitrogen). The cells were washed and resuspended in staining solution and filtered through a 35-μm filter (FALCON) before acquisition.

## Supporting Information

Table S1 **Serum Fc-fusion protein levels and hematological parameters in the EPOR-Fc Tg chimeras (8 weeks old).** The values are presented as the mean ±SD. ***, P<0.001 vs. Cont. (Student’s *t*-test).
(DOC)

Figure S1 **Diagram of the Igk locus of the WT mouse genome and the TG mouse in our previous work and this study.** 5’E : Igk intronic enhancer, 3’E : Igk 3’-enhancer, Puro: puromycin-resistant marker, Neo: G418-resistant marker, P: promoter, LS: leader sequence derived from the Igk region, RS: recombination sequence, pA: Igk-polyA, cDNA(-LS): the native LS coding region of cDNA was removed in this study.
The promoter sequence fragment used in a previous study (0.21kb) derived from the Igkv4-54 region and the promoter( 0.5kb)/leader sequence (0.3kb) fragment used in this study derived from the Igkv3-12 region were amplified via PCR using C57BL/6 mouse genomic DNA as a template with the following primer pairs:
previous study: Fw-CCCAAGCTTTGGTGATTATTCAGAGTAGTTTTAGATGAGTGCAT, Rv-ACGCGTCGACTTTGTCTTTGAACTTTGGTCCCTAGCTAATTACTA; .
this study: Fw-CCTTAATTAAAGTTATGTGTCCTAGAGGGCTGCAAACTCAAGATC, Rv-TTGGCCGGCCTTGGCGCCAGTGGAACCTGGAATGATAAACACAAAGATTA TTG.
(TIF)

Figure S2 **Histological images of mice exhibiting each of the phenotypes described in Figure 3B.** The arrows indicate the relevant areas of each phenotype. A. Brain (calcification), B. femur (increased trabecular bone (T)), C. heart (epicarditis), D. lung (pleuritis), E. spleen (fibrosis), F. rib (enlargement), G. thymus (fatty infiltration), H. duodenum (calcification), I. forelimb (vascular calcification in the digit), J. incisor of the mandible (hypoplasia of dentin) and K. testis (atrophy). As indicated, more spaces existed in Tg chimeras). Each scale bar indicates 200 μm.
(TIF)

Figure S3 **Increased muscle phenotype in the ActRIIB-Fc mice.** The 11-week-old ActRIIB-Fc mice exhibited significantly increased muscle volume compared to the control mice of the same age (A). In addition, the body weight was increased in the ActRIIB-Fc mice (B). Each scale bar indicates 1 cm.
(TIF)

Figure S4 **Histological images of the bone marrow obtained from each Tg chimera (8 weeks old).** A. Control, B ActRIIA-Fc, C. ActRIIB-Fc, D. BMPRII-Fc. There were no obvious differences in the frequency of foci of erythroblastic islets between the control chimeras and each Tg chimera. Each scale bar indicates 200 μm.
(TIF)

Figure S5 **Flow cytometric histograms of bone marrow and spleen of each chimeric mice.** A. ActRIIA-Fc, B.ActRIIB-Fc, C.BMPRII-Fc. The CD71+TER119+ populations of spleen are marked up with red circle. f : female, m : male.
(TIF)
