# Supplementary material for: Adult-Specific Systemic Over-Expression Reveals Novel In Vivo Effects of the Soluble Forms of ActRIIA, ActRIIB and BMPRII
Source: PLoS One. 2013 Oct 21;8(10):e78076. doi: 10.1371/journal.pone.0078076 (PMC3804470; doi:10.1371/journal.pone.0078076)
Supplement: Table S1 — Serum Fc-fusion protein levels and hematological parameters in the EPOR-Fc Tg chimeras (8 weeks old). The values are presented as the mean ±SD. ***, P<0.001 vs. Cont. (Student’s t-test). (DOC) [file pone.0078076.s001.doc]

**Table S1 The serum Fc-fusion protein level and hematological parameters of EPOR-Fc chimeras(8 week-old).**

|  | **EPOR-Fc** | | **Control** | |
| --- | --- | --- | --- | --- |
|  | **Female** | **Male** | **Female** | **Male** |
| **N** | **8** | **8** | **12** | **12** |
| **Serum protein level**  **[ug/ml]** | **25.5 ± 2.7** | **23.9 ± 2.4** | **N.D.** | **N.D.** |
| **RBC(^10^6^[cells/ul])** | **4.1 ± 0.74^***^** | **3.8 ± 0.62^***^** | **10.3 ± 0.40** | **10.1 ± 0.37** |
| **HGB([g/dL])** | **5.7 ± 1.02^***^** | **5.4 ± 1.05^***^** | **15.6 ± 0.32** | **15.9 ± 0.48** |
| **HCT([%])** | **20.5 ± 3.61^***^** | **19.5 ± 3.12^***^** | **52.9 ± 1.29** | **53.2 ± 1.73** |
| **Retic (^10^9^[cells/L])** | **148.9 ± 55.2^***^** | **95.2 ± 36.6^***^** | **250.8 ± 51.8** | **302.2 ± 45.2** |

**Values are given as the means ±SD. ***, P<0.001 vs. Cont. (student T-test)**
