# Supplementary figures and images for: Adult-Specific Systemic Over-Expression Reveals Novel In Vivo Effects of the Soluble Forms of ActRIIA, ActRIIB and BMPRII
Source: PLoS One. 2013 Oct 21;8(10):e78076. doi: 10.1371/journal.pone.0078076 (PMC3804470; doi:10.1371/journal.pone.0078076)

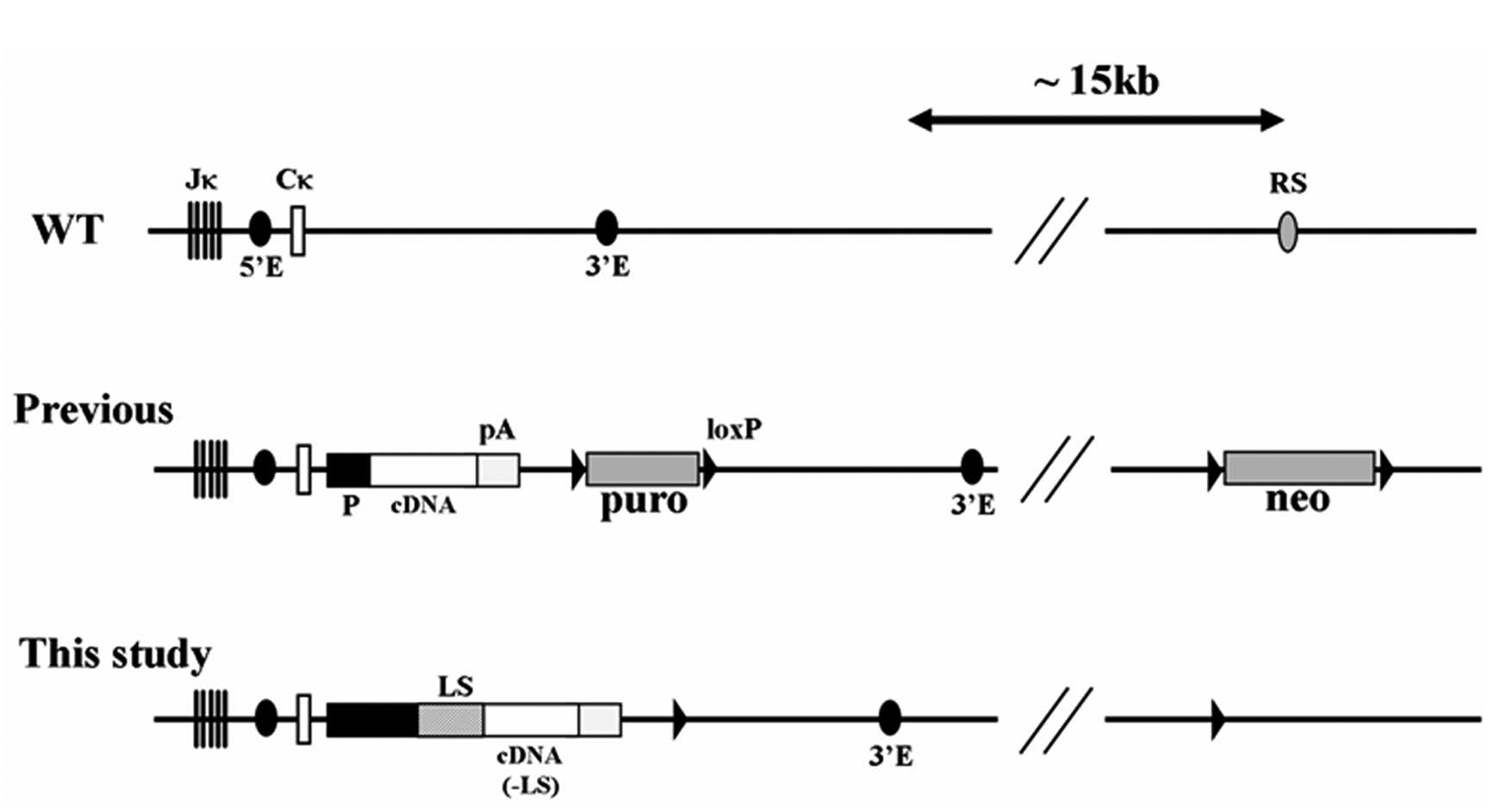

Supplement: Figure S1 — Diagram of the Igk locus of the WT mouse genome and the TG mouse in our previous work and this study. 5’E : Igk intronic enhancer, 3’E : Igk 3’-enhancer, Puro: puromycin-resistant marker, Neo: G418-resistant marker, P: promoter, LS: leader sequence derived from the Igk region, RS: recombination sequence, pA: Igk-polyA, cDNA(-LS): the native LS coding region of cDNA was removed in this study. The promoter sequence fragment used in a previous study (0.21kb) derived from the Igkv4-54 region and the promoter( 0.5kb)/leader sequence (0.3kb) fragment used in this study derived from the Igkv3-12 region were amplified via PCR using C57BL/6 mouse genomic DNA as a template with the following primer pairs: previous study: Fw-CCCAAGCTTTGGTGATTATTCAGAGTAGTTTTAGATGAGTGCAT, Rv-ACGCGTCGACTTTGTCTTTGAACTTTGGTCCCTAGCTAATTACTA; . this study: Fw-CCTTAATTAAAGTTATGTGTCCTAGAGGGCTGCAAACTCAAGATC, Rv-TTGGCCGGCCTTGGCGCCAGTGGAACCTGGAATGATAAACACAAAGATTA TTG. (TIF) [file pone.0078076.s002.tif]

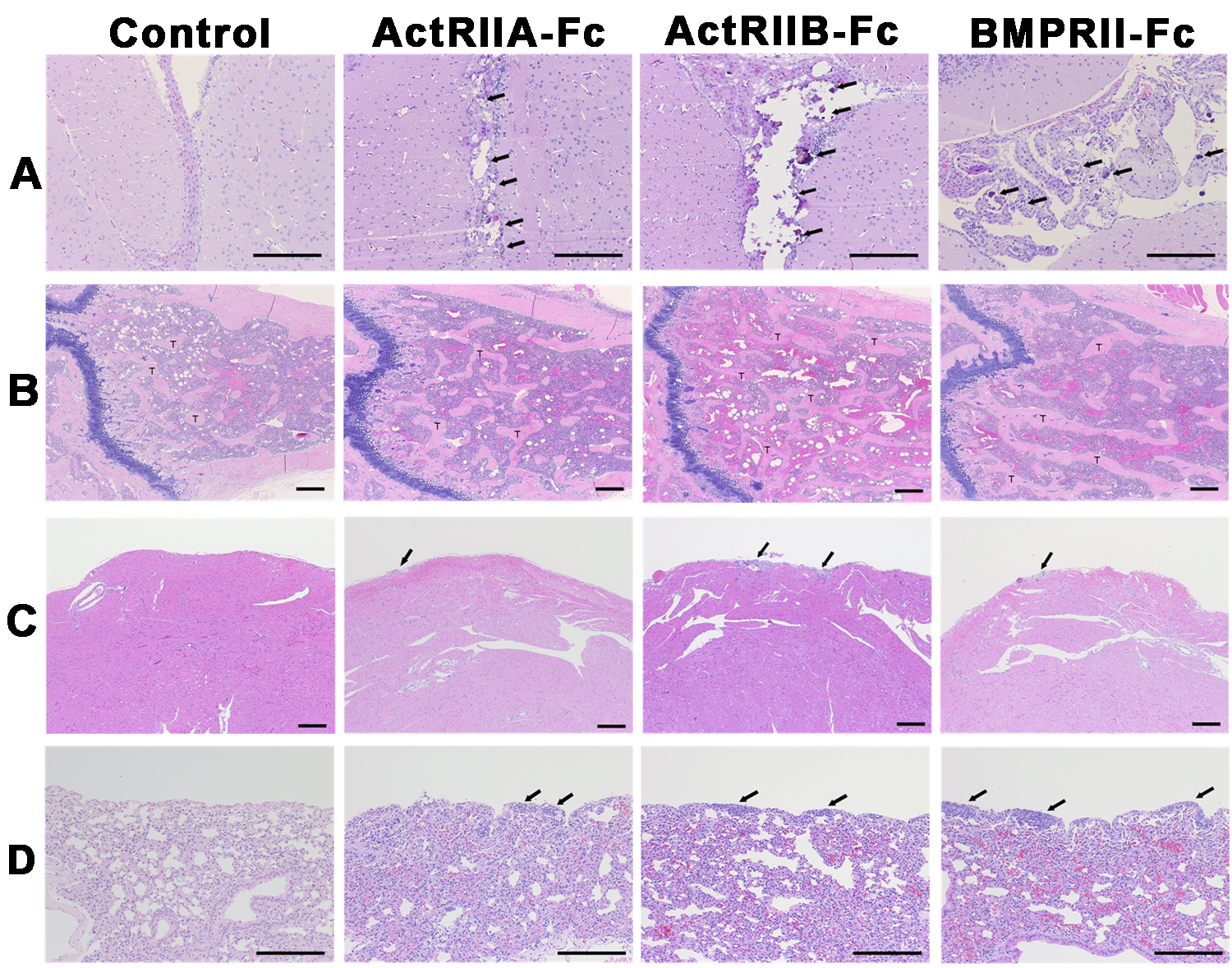

Supplement: Figure S2 — Histological images of mice exhibiting each of the phenotypes described in Figure 3B. The arrows indicate the relevant areas of each phenotype. A. Brain (calcification), B. femur (increased trabecular bone (T)), C. heart (epicarditis), D. lung (pleuritis), E. spleen (fibrosis), F. rib (enlargement), G. thymus (fatty infiltration), H. duodenum (calcification), I. forelimb (vascular calcification in the digit), J. incisor of the mandible (hypoplasia of dentin) and K. testis (atrophy). As indicated, more spaces existed in Tg chimeras). Each scale bar indicates 200 μm. (TIF) [file pone.0078076.s003.tif]

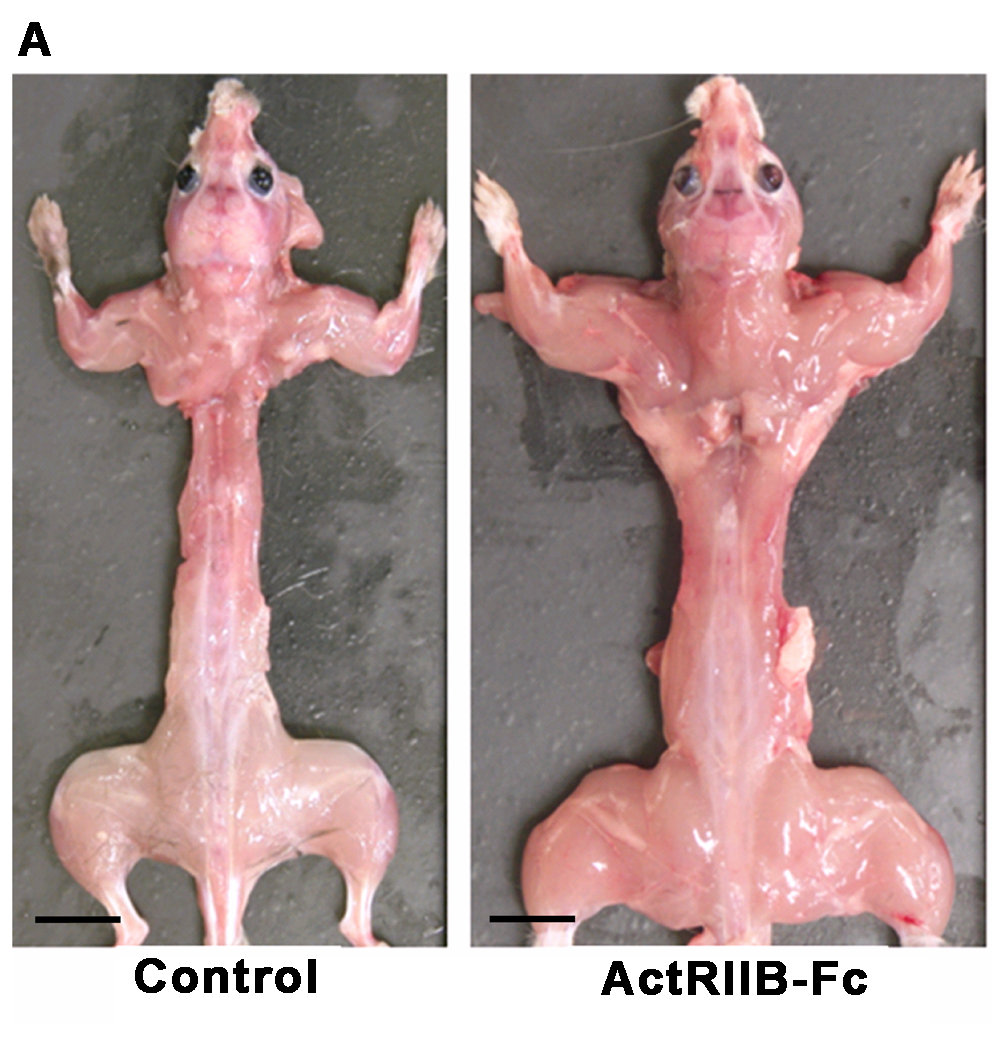

Supplement: Figure S3 — Increased muscle phenotype in the ActRIIB-Fc mice. The 11-week-old ActRIIB-Fc mice exhibited significantly increased muscle volume compared to the control mice of the same age (A). In addition, the body weight was increased in the ActRIIB-Fc mice (B). Each scale bar indicates 1 cm. (TIF) [file pone.0078076.s004.tif]

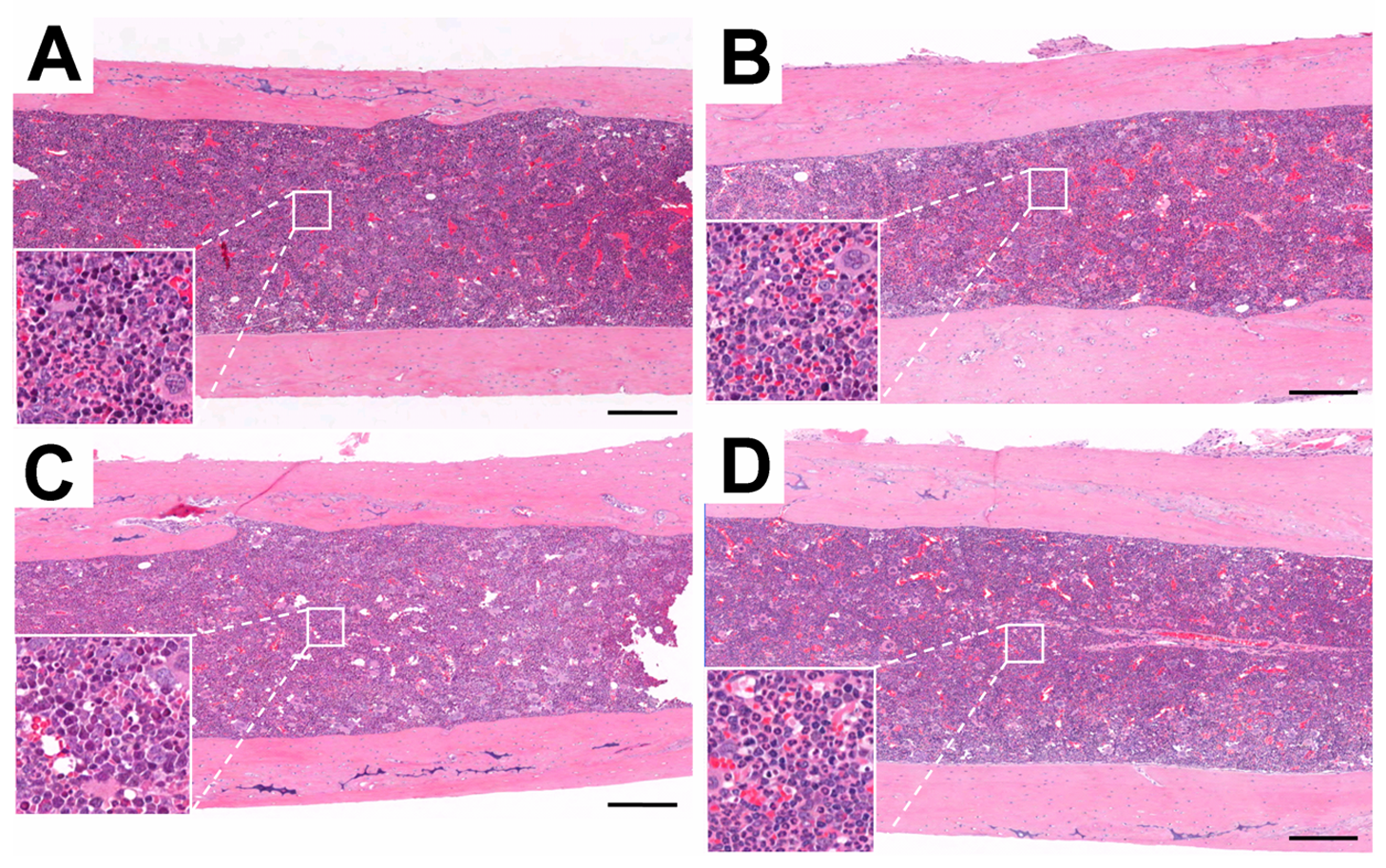

Supplement: Figure S4 — Histological images of the bone marrow obtained from each Tg chimera (8 weeks old). A. Control, B ActRIIA-Fc, C. ActRIIB-Fc, D. BMPRII-Fc. There were no obvious differences in the frequency of foci of erythroblastic islets between the control chimeras and each Tg chimera. Each scale bar indicates 200 μm. (TIF) [file pone.0078076.s005.tif]

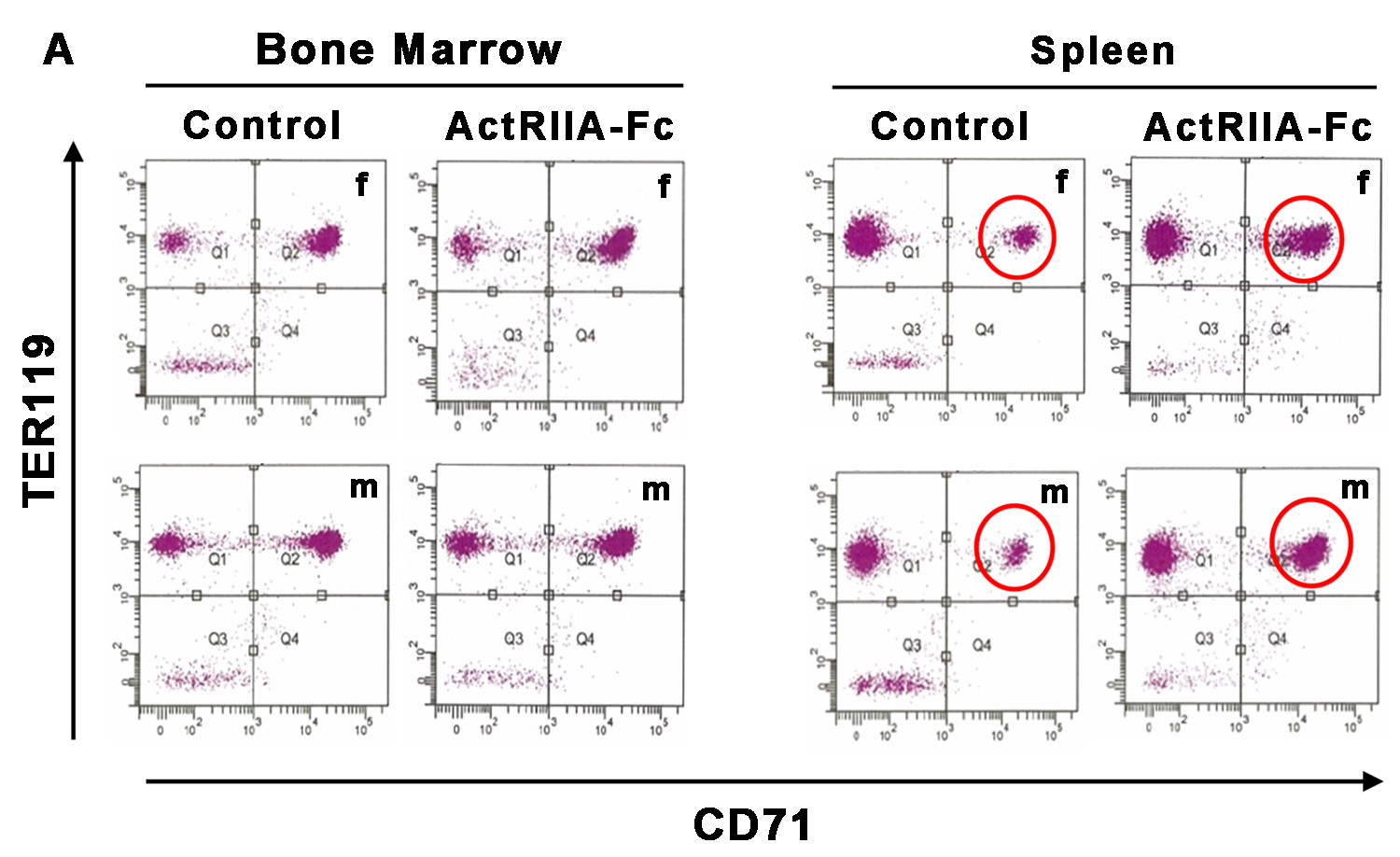

Supplement: Figure S5 — Flow cytometric histograms of bone marrow and spleen of each chimeric mice. A. ActRIIA-Fc, B.ActRIIB-Fc, C.BMPRII-Fc. The CD71+TER119+ populations of spleen are marked up with red circle. f : female, m : male. (TIF) [file pone.0078076.s006.tif]
